# Enriching the FIDEO ontology with food-drug interactions from online knowledge sources

**DOI:** 10.1186/s13326-024-00302-5

**Published:** 2024-03-04

**Authors:** Rabia Azzi, Georgeta Bordea, Romain Griffier, Jean Noël Nikiema, Fleur Mougin

**Affiliations:** 1 Univ. Bordeaux, Inserm, BPH, U1219, F-33000 Bordeaux, France; 2 CHU de Bordeaux, Service d’information médicale, F-33000 Bordeaux, France; 3 Univ. La Rochelle, L3i, F-17000 La Rochelle, France; 4 Department of Management, Evaluation and Health Policy, School of Public Health, Université de Montréal, Québec, Canada

**Keywords:** Biomedical ontology, Food-drug interactions, Adverse drug effects, FAIR principles

## Abstract

The increasing number of articles on adverse interactions that may occur when specific foods are consumed with certain drugs makes it difficult to keep up with the latest findings. Conflicting information is available in the scientific literature and specialized knowledge bases because interactions are described in an unstructured or semi-structured format. The FIDEO ontology aims to integrate and represent information about food-drug interactions in a structured way. This article reports on the new version of this ontology in which more than 1700 interactions are integrated from two online resources: DrugBank and Hedrine. These food-drug interactions have been represented in FIDEO in the form of precompiled concepts, each of which specifies both the food and the drug involved. Additionally, competency questions that can be answered are reviewed, and avenues for further enrichment are discussed.

## Introduction

Food-drug interactions, such as the well-known interaction with grapefruit juice [1], should be extensively documented and appropriately acknowledged by patients and medical professionals because they may have serious consequences on the outcome of treatments and can be life-threatening. Indeed, a decrease in expected drug effects or an increase in toxicity are particularly problematic for vulnerable people, such as patients with cancer, transplant recipients, or HIV-positive individuals [2]. As the challenge is that articles reporting these interactions become more prevalent [3], the important opportunity arises to mine the literature to be able to represent and make readily available current knowledge about food-drug interactions. This was the objective of the French research project MIAM [4] which ended in 2021. Within the framework of this project, natural language processing methods were developed to extract entities (food and drugs, in particular) and relations between these entities [5], as well as the Food Interactions with Drugs Evidence Ontology (FIDEO) in order to model knowledge about food-drug interactions [6].

The first version of FIDEO was developed following the METHONTOLOGY methodology [7] using an essentially manual process. The approach followed was modular, with the reuse of several ontologies describing foods (FoodOn [8]), drugs (Chemical Entities of Biological Interest, ChEBI [9]), interactions between drugs (Drug-drug Interaction and Drug-drug Interaction Evidence Ontology, DIDEO [10]), and information entities that represent the scientific source of interactions (Information Artifact Ontology, IAO [11]). The high-level ontology Basic Formal Ontology (BFO) [12] is used to define FIDEO according to a global interoperability framework. A preliminary population of FIDEO was initially manually performed for test purposes, using a few abstracts annotated by pharmacology experts.

For the new version of FIDEO, we closely followed FAIR (Findable, Accessible, Interoperable, Reusable) principles that describe how data should be organized to be more easily accessible, understood, exchangeable, and reusable [13]. These principles not only apply to research data but also to the tools, algorithms, and workflows that lead to the results. This aids to enhance transparency, reproducibility, and reuse of research outcomes [14]. The process of building an ontology can be automated to support semantic interoperability, which is essential according to FAIR principles to promote collaboration and openness. In this context, the Open Biomedical and Biological Ontologies (OBO) Foundry [15], launched in 2007 to provide a framework in the form of a set of principles and tools for ontology development and maintenance, was a forerunner. Indeed, the uncontrolled and very rapid development of numerous ontologies in the biological domain at the beginning of the 2000s made it necessary to implement an integrative and interoperable approach. The principles originally defined by the OBO Foundry were theoretical and subject to interpretation. To ensure that the ontologies referenced by OBO are FAIR, operational rules and tools have recently been implemented to automatically validate the compliance of these ontologies with OBO principles [16].

The purpose of this article is thus to present the new version of FIDEO; and more precisely the evolution of the initial model, the implementation of an automatic process for enriching the ontology from external knowledge sources and the competency questions that are now possible to answer. To maximize interoperability and usability, we followed a methodology that aims to develop ontologies complying with the FAIR principles. In addition, the model proposed in the first version has been simplified to allow the ontology to be automatically enriched and queried. The coverage and the reliability of food-drug interactions described in FIDEO has been greatly increased by integrating external knowledge from expert-curated databases instead of information derived directly from scientific articles. The new version of FIDEO is available at: https://gitub.u-bordeaux.fr/erias/fideo/.

## Related work

Existing ontologies represent knowledge related to food-drug interactions. The ontology most closely related to FIDEO is DIDEO, an ontology for describing potential drug-drug interactions [10]. It adopts an evidence-based approach to representing drug-drug interactions, recognizing the need to supply users with evidence that enables them to assess the clinical significance of an interaction and make decisions [17]. The type of study depicting an interaction is also modeled in the ontology, including *in vitro* experiments, population pharmacokinetic analyses, and observational epidemiological studies. DIDEO was then extended to integrate interactions between drugs and natural products such as herbal supplements [18]. Another ontology describing drug-drug interactions is DINTO, the Drug-Drug Interactions Ontology [19]. However, this ontology was not designed from the outset on the basis of BFO (although an alignment of concepts on BFO was subsequently proposed) and it has not been updated since 2015. With regards to drugs, the two most widely used ontologies are: (i) DrOn, the Drug Ontology [20] which is a modular and extensible ontology of drug products, their ingredients and biological activity, and (ii) ChEBI, an ontology on chemical entities of biological interest [9]). Finally, large-scale efforts have been underway for several years to model the food domain in the ontology FoodOn [8, 21]. This is a large and growing resource on food that is regularly enriched, for example by information about food types contained in Wikipedia [22]. In addition, FoodOn has been extended by the Ontology for Nutritional Epidemiology (ONE) with nutritional epidemiology concepts, to assesses the links between diet, nutrients and health, as well as disease outcomes [23].

Limited information about food-drug interactions is available in a clinical setting as these interactions are mostly described in scientific publications and disparately in generic databases about drugs such as DrugBank. Current efforts to structure this information in a dedicated database using natural language processing result in a very large number of potential interactions that are less reliable [24]. For instance, recent results on the Drug-Food Interaction (DFI) corpus place the performance of automated extraction from scientific publications at only 55% F-score [25]. High-quality information on food-drug interactions is particularly important in the context of advanced visualisations that aim to provide an overview of this field and enable exploration and discovery [26]. It is also necessary for developing graph embedding approaches to identify potential food-drug interactions [27]. In this work, we aim to reduce noise by relying on information manually curated by domain experts in existing databases.

In order to increase interoperability, close attention is given to integration with other ontologies from the food domain [21] and following the principles of the “eXtensible Ontology Development” (XOD) methodology. This methodology is focused on four main principles that should be followed in order to develop extensible and interoperable ontologies [28]. These principles provide guidelines around term reuse (XOD 1), semantic alignment (XOD 2), design pattern usage (XOD 3), and community extensibility (XOD 4). Following these principles, CIDO [29] and ICDO [30] were designed to describe different facets of COVID-19. CIDO developers explain in detail how they implemented each principle of the XOD strategy recursively to continually enrich the ontology as more is known. In contrast, ICDO authors do not provide much information about how they applied the XOD principles. An ontology representing the knowledge about vaccine studies, Vaccine Investigation Ontology (VIO), was also recently developed following the XOD principles [31]. The authors do not explicitly refer to the four principles in their description of the ontology design methodology followed but they mention the use of the OntoFox tool [32] to address principles XOD 1 and XOD 2 and define ontology design patterns (related to XOD 3). In addition, a use case investigating the possibility of representing the data described in yellow fever vaccine studies using VIO is consistent with the XOD 4 principle. Finally, other recently published articles also briefly describe their use of XOD principles in developing their ontology [33, 34].

It should be noted that all five articles describing the ontologies cited above involve the first author of the article that presents the XOD methodology. A contribution of our work is to report our experience in following this methodology using only publicly available documentation, without any direct input from the XOD authors. Last year, the OBO community has released the ODK toolkit with the same commitment to standardization as XOD but also with automated workflows to help ontology developers in their tasks [35]. ODK did not offer these features at the time we developed FIDEO, so we did not consider it, but the desired convergence of these two approaches should allow us to benefit from the functionalities provided by ODK to keep respecting the principles of XOD in future developments of FIDEO.

In previous work, we developed the FIDEO ontology [6] which is to our knowledge the only one dedicated to food-drug interactions. The first version was composed of 739 concepts, 108 relationships (i.e. object properties). Only 33 concepts were specific to food-drug interactions and had thus to be created. The remaining 706 concepts have been imported from other existing ontologies (namely BFO, DIDEO, FoodOn, ChEBI, IAO). In this paper, we present how the initial FIDEO model has evolved along with an automatic approach developed to enrich the ontology with food-drug interactions acquired from two online knowledge resources. To ensure that FIDEO is FAIR, we used the XOD methodology and we detail here the different steps followed to comply with its four principles.

## Materials

In this section, we describe: (i) online resources providing the information we integrated regarding food-drug interactions, (ii) the ATC classification used for structuring drugs in FIDEO, (iii) the tools we used to add new concepts in FIDEO.

### Resources containing food-drug interactions

#### DrugBank

We used the 5.0 version of DrugBank [36], containing “*comprehensive molecular information about drugs, their mechanisms, their interactions and their targets*” [37]. This release includes new data, improves the quality and consistency of all existing drug indications, and enhances the information related to drug-drug and food-drug interactions. In DrugBank 5.0, the number of drugs has been increased from 1836 to 2358. However, information on food-drug interactions is still available in the form of a brief text description. As an example, DrugBank asserts treatment recommendations for five food interactions for “warfarin” described as “*Avoid drastic dietary changes.*”, “*Avoid foods rich in vitamin K. Vitamin K in foods such as leafy vegetables can reduce warfarin efficacy.*”, “*Avoid grapefruit products. They may interfere with warfarin metabolism and increase INR, increasing the risk of bleeding.*”, “*Avoid herbs and supplements with anticoagulant/antiplatelet activity. Examples include garlic, ginger, bilberry, danshen, piracetam, and ginkgo biloba.*” and “*Avoid St. John’s Wort. This drug may reduce warfarin efficacy.*”.

#### Hedrine

Since medicinal and aromatic plants used for medical treatment or human consumption are an important part of food systems, we have decided to integrate herb-drug interactions in FIDEO. These alternative treatments are increasingly popular, especially for cancer patients and during pregnancy, but awareness of their side effects is limited because people perceive them to be more natural. Hedrine [38] is a widely used knowledge resource available in French, that lists clinical studies and case reports about interactions between medicinal plants and allopathic drugs (159 plants, 604 drugs and 3743 interactions) [39]. Also described are potential interactions via pharmacodynamic or pharmacokinetic mechanisms. In addition, Hedrine provides high-quality, manually curated and structured data that is freely available for health professionals (provided an account is created).

### ATC for structuring drugs hierarchically

In the Anatomical Therapeutic Chemical (ATC) classification system, active substances are divided into different groups according to the organ or system on which they act and their therapeutic, pharmacological and chemical properties. Drugs are classified into groups at five different levels. To provide a general and simple hierarchy of drugs involved in food-drug interactions, we used the first two levels of ATC classes. The first level structures the drugs according to major anatomical or pharmacological groups and the second level according to pharmacological or therapeutic subgroups. It should be noted that some drugs can be categorized in several classes because they have multiple indications. For example, “heparin” belongs to the following subgroups: “B01 Antithrombotic agents” (B being the Blood and blood forming organs), “C05 Vasoprotectives” (C being the Cardiovascular system), and “S01 Ophtalmologicals” (S being the Sensory organs).

### Tools for enriching FIDEO

#### Ontology lookup service

In order to integrate concepts from existing ontologies in accordance with the FAIR principles, it is necessary to query multiple domain-specific ontologies and controlled vocabularies through interactive and programmatic means. The Ontology Lookup Service (OLS) [40], developed and maintained by the Samples, Phenotypes and Ontologies Team (SPOT) at EMBL-EBI, is a repository for biomedical ontologies that provides a single web-based access point to the latest versions of these ontologies [41]. Ontologies can be browsed through the website as well as programmatically via the SPOT API.

#### ROBOT

To promote the adoption of the FAIR principles, the OBO Foundry developed ROBOT (a recursive acronym for “ROBOT is an OBO Tool”) that is an open source library and a command-line tool for automating ontology development tasks [42]. ROBOT provides ontology processing functionalities for a variety of tasks, such as converting formats, running a reasoner, creating importable modules, running reports. These facilities can be combined into larger workflows using a separate task execution system, such as GNU make, and the workflows can be executed automatically in continuous integration systems.

## Methodology

This section presents: (i) the modifications that have been made to the model of the first version of FIDEO, (ii) the enrichment process of FIDEO to include food-drug interactions from DrugBank and Hedrine, and (iii) the evaluation framework with regards to competency questions.

### Evolution of the FIDEO model

The first version of FIDEO focused mainly on how to organize knowledge related to food-drug interactions, and in particular how to link together existing concepts (from external ontologies) and new ones (defined in FIDEO), but not on the actual interactions described in the literature between food and drugs. In this new version, we represent food-drug interactions listed in online resources but currently described in a non-formalized way.

**Fig. 1:**
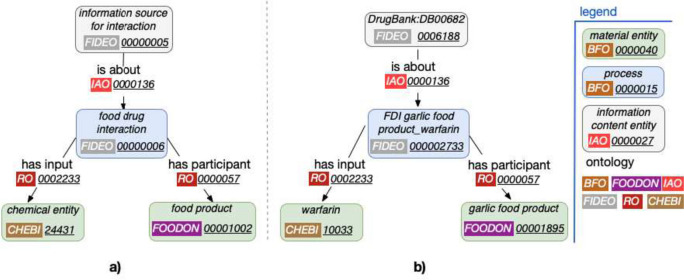
**a** Organization of high-level concepts related to the food-drug interaction process in FIDEO, and **b**) Illustrative example of this framework with a food-drug interaction between garlic and warfarin described in DrugBank at the following URL: https://go.drugbank.com/drugs/DB00682. The ontologies from which the corresponding concepts and the relations existing between them originate, as well as the hierarchy to which these concepts belong are specified in the legend on the right of the figure

Compared to the model proposed in Fig. 1 of our previous paper [6], we have greatly simplified the Data part, corresponding to information derived from scientific knowledge. We use only the newly created concept “information source for interaction” (child concept of “information content entity” (IAO:0000030)), which is the parent concept of all evidence describing a given food-drug interaction and is linked to the process of that interaction by the relationship *is_about* (Fig. 1a). Food-drug interactions have been represented in FIDEO in the form of precompiled concepts, each of which specifies both the food and the drug involved. More specifically, the process of the interaction is linked to the food concerned (material entity) by the relationship *has_participant* and to the drug affected (material entity) by the relationship *has_input*. The definition of these relations are as follows:

- *has_participant*: “*a relation between a process and a continuant, in which the continuant is somehow involved in the process*” (source: http://purl.obolibrary.org/obo/RO_0000057),
- *has_input*: “*p has input c if and only if p is a process, c is a material entity, c is a participant in p, c is present at the start of p, and the state of c is modified during p*” (source: http://purl.obolibrary.org/obo/RO_0002233).

In the food-drug interactions described in the Hedrine and DrugBank resources, the information we have (and therefore represent) concerns the change that occurs in the effect of a given drug in the presence of a given food. Thus, both the food and the drug are involved in the interaction, but we consider that only the state of the drug is altered during this interaction (implying the use of the *has_input* relationship, being more specific than *has_participant*). Finally, the Real world part, which describes information about biomedical entities, has also been simplified and the “chemical substance” (ChEBI:59999) concept has been replaced by its parent concept “chemical entity” (ChEBI:24431) because many drugs found in DrugBank are classified as “chemical entity” in ChEBI, not as “chemical substance”.

Figure 1 illustrates the main concepts involved in describing a food-drug interaction in the second version of the model of FIDEO (panel a), and how an interaction between garlic and warfarin listed in DrugBank is represented in the ontology (panel b).

### FIDEO enrichment steps and design aspects

**Fig. 2:**
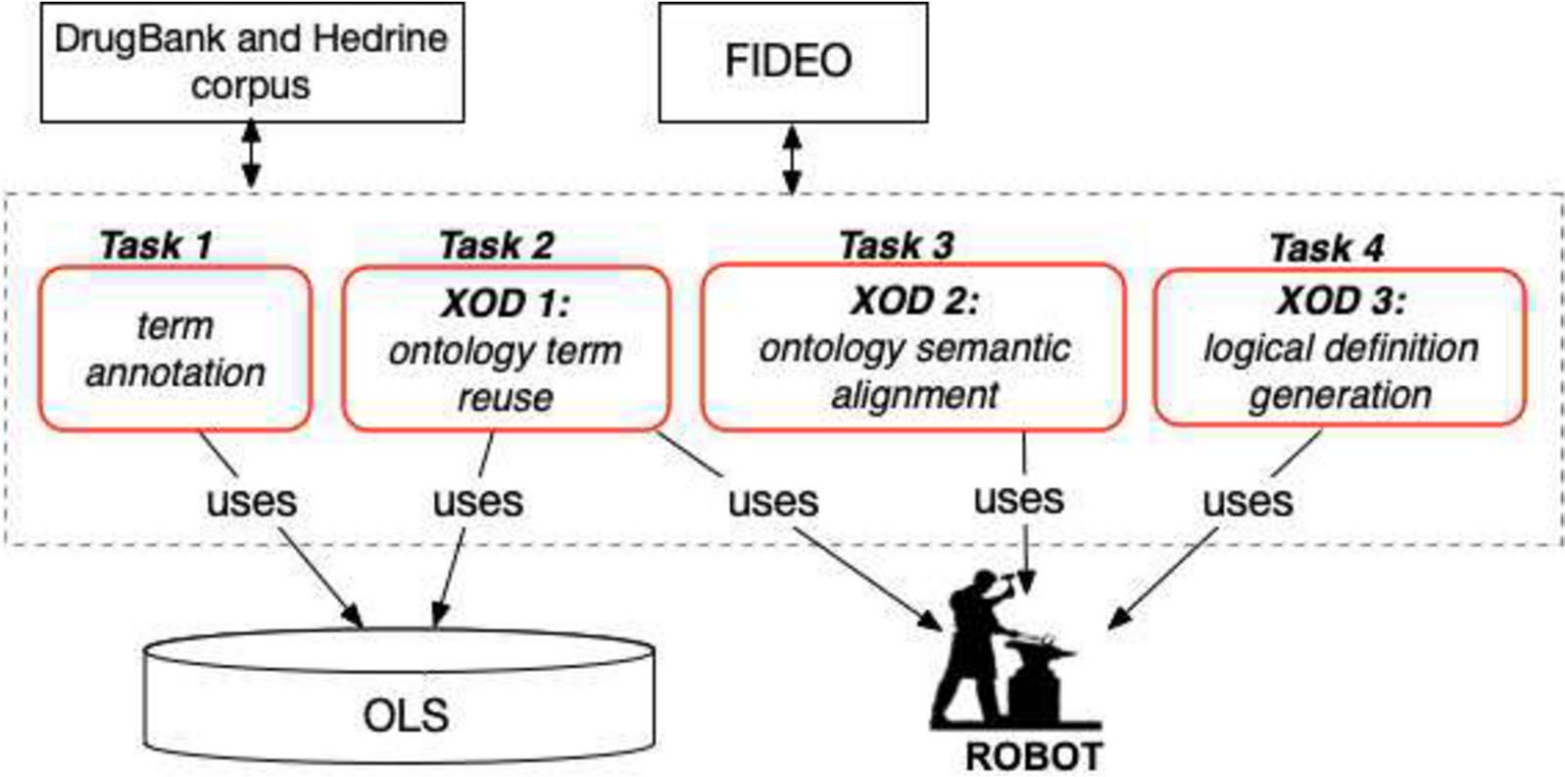
Complete FIDEO enrichment process. Knowledge was extracted from DrugBank and Hedrine, OLS was used to identify drugs, foods and herbs in existing ontologies and ROBOT to define patterns for creating logical definitions for concepts to be included in FIDEO

In this part, we present the general architecture of the approach implemented to enrich FIDEO, which consists of the following four tasks shown in Fig. 2: 1) *term annotation* of food and herb terms within the DrugBank food-drug interaction corpus, 2) *ontology term reuse* (XOD 1) of concept names from existing ontologies to be mapped to annotation terms, 3) *ontology semantic alignment* (XOD 2) of selected concepts within FIDEO, and 4) *ontology design pattern usage for the generation of logical definitions for concepts* (XOD 3) to be integrated in FIDEO.

#### Task 1: term annotation

Textual descriptions provided in DrugBank were manually annotated to structure food-drug interactions. From the drugs with relevant information, we first annotated a subset of interactions involving 100 randomly selected drugs. This first corpus was annotated by three annotators (GB, RA, and FM) to check inter-annotator agreement for categories including Food, Food Component, Herb, Enzyme, Interaction Mechanism, Physiological Effect, and Meal Time. A total of 473 sentences was annotated in this way. In general, agreement was in the almost perfect range for all categories, with a Fleiss Kappa of 0.87 for the Food category and higher for the other categories. To ensure high coverage, food interactions with the remaining drugs were exhaustively annotated by two annotators (GB and RA). Each annotated half of these drugs and the third annotator (FM) finally reviewed all drugs to homogenize the annotation. Overall, a total of 1213 sentences have been annotated following this procedure. An illustration of the annotation of the five food interactions concerning “warfarin” is presented in Table 1.

**Table 1:** Annotations of the field *Food interactions* described in DrugBank for the drug “warfarin” (DrugBank:DB00682)

*Food Interactions* field	Food	Herb
Avoid drastic dietary changes. Avoid foods rich in vitamin K. Vitamin K in foods such as leafy vegetables can reduce warfarin efficacy. Avoid grapefruit products. They may interfere with warfarin metabolism and increase INR, increasing the risk of bleeding. Avoid herbs and supplements with anticoagulant/antiplatelet activity. Examples include garlic, ginger, bilberry, danshen, piracetam, and ginkgo biloba. Avoid St. John’s Wort. This drug may reduce warfarin efficacy.	foods rich in vitamin K, leafy vegetables, anticoagulant / antiplatelet supplements, grapefruit products	anticoagulant / antiplatelet herbs, garlic, ginger, bilberry, danshen, piracetam, ginkgo biloba, St. John’s Wort

As food-drug interactions are fully structured in the Hedrine database, no additional annotation was required. Hedrine curators make use of dedicated fields for foods, plants, drugs, interaction mechanisms and clinical importance that can be directly integrated in FIDEO.

#### Task 2: ontology term reuse

The principle of this task was to find a correspondence between the food, herb and drug terms used to annotate DrugBank sentences or described in Hedrine and concept names coming from existing ontologies. We automated this task to facilitate further enrichment of the ontology in future versions.

To perform these matches, we used the OLS service (described in Tools for enriching FIDEO section) to search for concepts in reference biomedical ontologies.

Since the terms to be searched in our case were related to the drug, food and herb categories, we performed term matching as follows: (i) drug terms were searched in ChEBI and, if not found, in the Drug Ontology (DrOn); (ii) food terms were searched in FoodOn; (iii) herb terms were searched in FoodOn and, if not found, in DrOn.

For each category, we estimated this automatic term mapping using the ratio of the number of terms mapped to a concept from existing ontologies to the total number of terms. At the end of this step, we found that the matching step was as follows: (1) Drugs: 1026 out of 1177 (87%) and 702 (59%), respectively for ChEBI and DrOn; (2) Foods: 54 out of 121 (45%) for FoodOn; (3) Herbs: 3 (20%) and 6 (40%) out of 15, respectively for FoodOn and DrOn. Because the automatic process was insufficient, we performed term matching manually for unmatched terms.

#### Task 3: ontology semantic alignment

This task aimed to organize concepts coming from ChEBI, FoodOn, and DrOn in a coherent way within FIDEO.

**Fig. 3:**
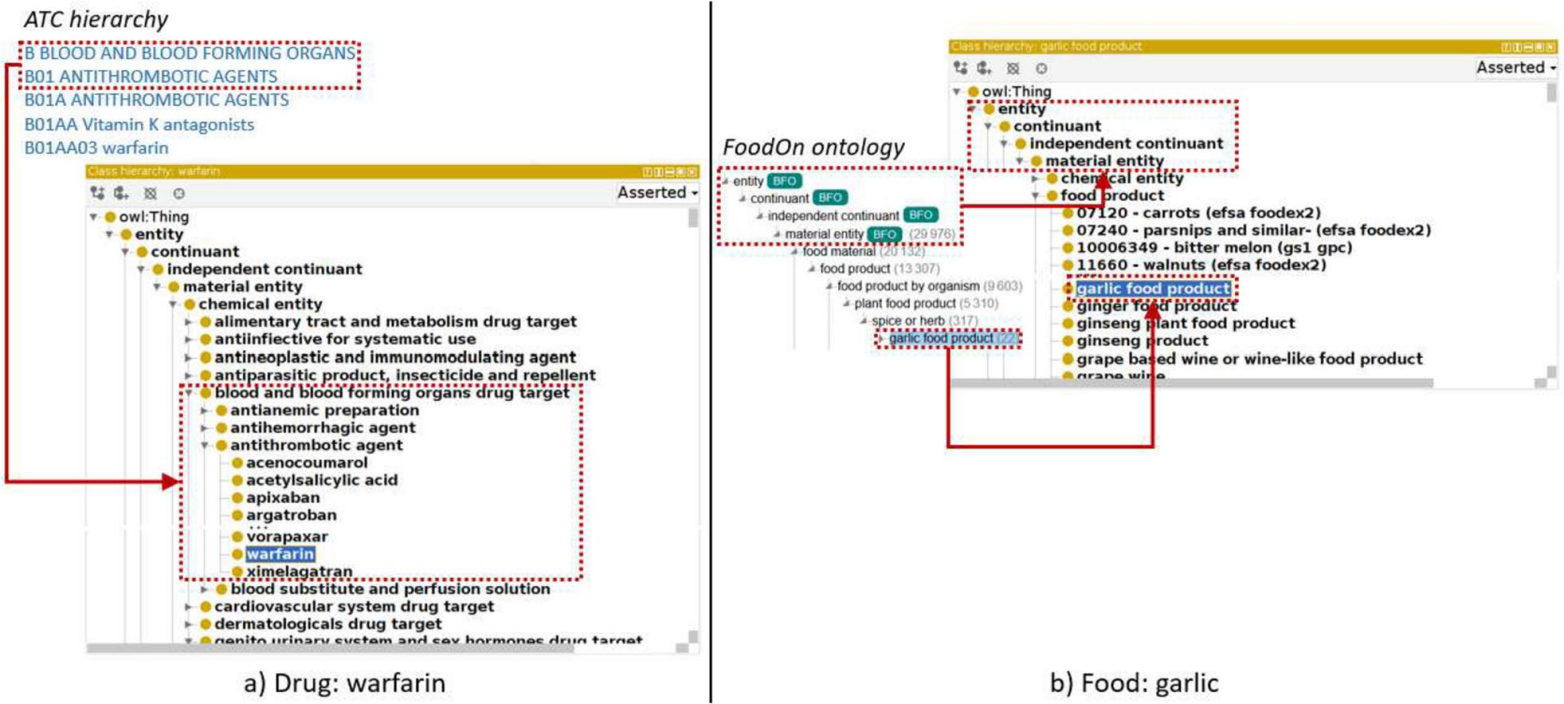
Integration of drugs and foods in FIDEO: **a** drugs are organized according to the ATC hierarchy (or as a “not elsewhere classified drug” for drugs not described in ATC), and **b**) foods are subclasses of the FoodOn concept “food product”

All drugs were described as sub-concepts of “chemical entity” (ChEBI:24431). To provide a simple and widely used structuration of drugs, we extracted for each drug obtained from DrugBank and Hedrine the ATC classes to which it belongs. We chose to integrate only the drug classes of the first two levels of the ATC (as illustrated in Fig. 3a)). To comply with OBO principles, we have converted plural terms to singular and lower-cased drug class names. In addition, we have slightly modified names that do not correspond to a drug type, and we have included the ATC code via a hasDbXref annotation containing the link to the web page corresponding to this ATC code. For example, the ATC class A-ALIMENTARY TRACT AND METABOLISM has been converted to FIDEO:00000100 with the label “drug target of alimentary tract and metabolism” and https://www.whocc.no/atc_ddd_index/?code=A DbXref. Finally, the drugs without ATC code were placed under an intermediate concept “not elsewhere classified drug” (e.g. “ardeparin” (DrOn:00017208)).

All foods for which a mapping in FoodOn was found ware integrated under the concept “food product” (FoodOn:00001002), which was positioned directly under the “material entity” (ignoring the intermediate concept “food material” (FoodOn:00002403)). Moreover, for the sake of simplification, we decided not to keep the sub-concepts described in the FoodOn hierarchy between “food product” and the foods actually involved in the food-drug interactions.

Herbs were integrated in the same way as foods for those that exist in FoodOn. For the other herbs (being associated with a DrOn concept), they were integrated under “processed material” as defined in DrOn.

Figure 3 illustrates the integration of the drug “warfarin” from ChEBI (panel a) and the food “garlic food product” from FoodOn (panel b).

#### Task 4: logical definition generation

For this task, we used a strategy based on ontology design patterns to assign logical definitions to concepts in FIDEO. This task complements the previous tasks by providing a specific, feasible and robust mechanism to obtain an easily maintainable ontology. For this purpose, we have implemented an approach based on the ROBOT tool, which allows generating an ontology in the Web Ontology Language (OWL) according to templates that can be used to develop modular ontologies. The advantage of this approach is that each module is associated with a spreadsheet, in which curators can update information about the ontology entities rather than directly editing the OWL ontology file. Then, ROBOT creates logical definitions and class annotations via the “template” command, which transforms the information described in a spreadsheet into OWL axioms.

**Fig. 4:**
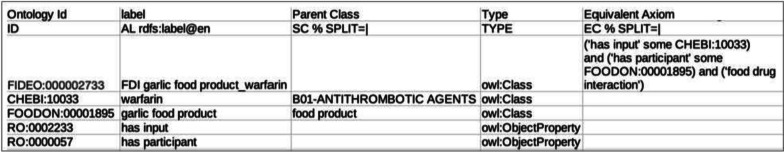
Extract from the ROBOT file containing as many rows as concepts and relations in FIDEO for which the following characteristics are available in the five columns: 1) their identifier in the ontology from which they are derived, 2) their label, 3) their parent concept(s) or relation(s) in FIDEO, 4) their type (owl:Class or owl:ObjectProperty), and 5) the logical definition(s) of defined concepts. Note that the concept “FDI garlic food product–warfarin” does not have a parent concept specified in column 3 because its logical definition already states that it is a child of the “food drug interaction” concept

Thus, we first built the template according to the model defined in Evolution of the FIDEO model section. In this template (Fig. 4), the first two rows represent the ROBOT header for specifying the entities and their properties (i.e. identifier, label, parent concept(s) if no equivalent axiom is provided, type - concept or relation - and equivalent axiom - or logical definition - of concepts). The rest of the file contains the concepts and relations of FIDEO. This approach allows to considerably reduce the time needed to integrate concepts, thus facilitating the ontology update process.

### Evaluation according to competency questions

In order to assess the coverage of the ontology, the competency questions that were defined for the first version of FIDEO are listed below, focusing on the interaction between garlic and warfarin used as an illustration throughout this article.

1. What foods potentially interact with warfarin?
2. Which drugs potentially interact with garlic?
3. Which antithrombotic agents may interact with garlic?
4. What type of interaction mechanisms underlie the interaction between garlic and warfarin?
5. What type of studies describe the interaction between garlic and warfarin?
6. What is the level of clinical importance of the garlic - warfarin interaction?
7. Which spices or herbs can be safely consumed by patients taking warfarin?
8. What alternative drugs can be taken to avoid the interactions between warfarin and garlic?

## Results

### Extraction from DrugBank and Hedrine

After parsing DrugBank data and keeping only those drugs for which the field *Food interactions* existed, 1177 drugs were extracted. After splitting the food-drug interaction textual descriptions into sentences for each drug involved, we obtained a total of 3759 food-drug interaction sentences. It is worth mentioning that when manually annotating interactions, we noticed that many sentences did not actually describe what we consider to be food-drug interactions, but in fact the absence of an interaction. For example, the DrugBank sentences describing food interactions with “alfacalcidol” are the following: “*Take with or without food. Food does not affect the bioavailability*”. For these cases, no annotation was made. In contrast, as soon as the sentence “*Take on an empty stomach*” appeared (e.g. “nafcillin”), the general annotation we chose was “food”. Once manual annotation of the remaining sentences has been completed, there were finally 1799 food-drug interactions involving 710 distinct drugs, 113 foods, and 25 herbs.

From Hedrine, 657 food-drug interactions have been acquired, involving 166 distinct drugs and 60 herbs.

### Integration of food-drug interactions in FIDEO

Overall, 613 distinct drugs, 76 foods and herbs from the DrugBank corpus have been mapped to at least one reference ontology - resulting in a total of 1245 food-drug interactions. Regarding the Hedrine corpus, 163 distinct drugs and the 60 herbs could be mapped to a concept in an existing ontology - resulting in a total of 654 food-drug interactions. Hedrine thus allowed the inclusion of interactions covering more diverse foods and herbs. Note that the distinction between herbs and drugs is not available in FIDEO, except in cases where the herbs had no match in FoodOn, which corresponds to 26 herbs (e.g. “licorice” (DrOn:00018111)).

Overall, the number of concepts integrated in FIDEO is therefore as follows:

- drugs: 721,
- foods and herbs: 138,
- food-drug interactions: 1702,
- information sources for interaction: 885 (662 from DrugBank and 223 from Hedrine).

It is quite normal that the global number of food-drug interactions (i.e. 1702) is lower than the sum of those described in DrugBank and Hedrine (i.e. 1245 + 654 = 1899) since some of them are described in both knowledge sources. This is the case for example for the interaction between garlic and warfarin. Furthermore, it was expected to have fewer concepts regarding the origin of the interaction described (i.e. 885) than the food-drug interactions themselves (i.e. 1702). Indeed, as illustrated in Fig. 1 and Table 1, DrugBank describes multiple food-drug interactions, such as that with garlic (“FDI garlic food product–warfarin” (FIDEO:000002733)) and with grapefruit (“FDI grapefruit food product–warfarin” (FIDEO:000002276)) as can be seen on the “warfarin” web page (which itself corresponds to the unique concept “DrugBank:DB00682” (FIDEO:0006188) of type “information source for interaction”).

In terms of drug articulation with respect to the ATC, the 14 top ATC drug classes were integrated in FIDEO (meaning that at least one drug from each main anatomical group is involved in a food-drug interaction) and 70 drug classes of the second level, out of a total of 94 in ATC. Then, 590 distinct drugs with an ATC code (e.g. “heparin”) and 137 distinct drugs without an ATC code (e.g. “ardeparin”) are included in FIDEO.

Statistics on foods and drugs involved in food-drug interactions are provided in Table 2. Foods involved in such interactions interact with an average of 12.2 drugs and a maximum of 326 interactions for “alcoholic beverage”, as illustrated in Table 3. On the other hand, drugs involved in such interactions interact with an average of 2.3 foods and a maximum of 17 interactions for “cyclosporin” and “warfarin”, as illustrated in Table 4.

**Table 2:** Statistics on foods and drugs involved in food-drug interactions as described in DrugBank and Hedrine

	Mean	Q1	Median	Q2	Maximum	Count
**Foods**	12.2	1	2	5	326	138
**Drugs**	2.3	1	1	3	17	721

**Table 3:** The 10 foods most frequently involved in food-drug interactions and their occurrence

Food	Count
alcoholic beverage	326
hypericum perforatum	254
grapefruit food product	168
food product	117
turmeric food product	96
glycyrrhiza glabra extract	63
ginkgo biloba extract	48
garlic food product	46
ginger food product	36
milk thistle extract	33
Total (percentage)	1187 (70%)

**Table 4:** The 11 drugs most frequently involved in food-drug interactions and their occurrence

Drug	Count
cyclosporin	17
warfarin	17
paracetamol	15
glyburide	13
trioxsalen	12
bortezomib	11
phenelzine	11
gliclazide	10
levothyroxine	10
tranylcypromine	10
verapamil	10
Total (percentage)	136 (8%)

### Competency questions in FIDEO

We detail here the ability of the new version of FIDEO to answer the eight competency questions listed in Evaluation according to competency questions section.

**Fig. 5:**
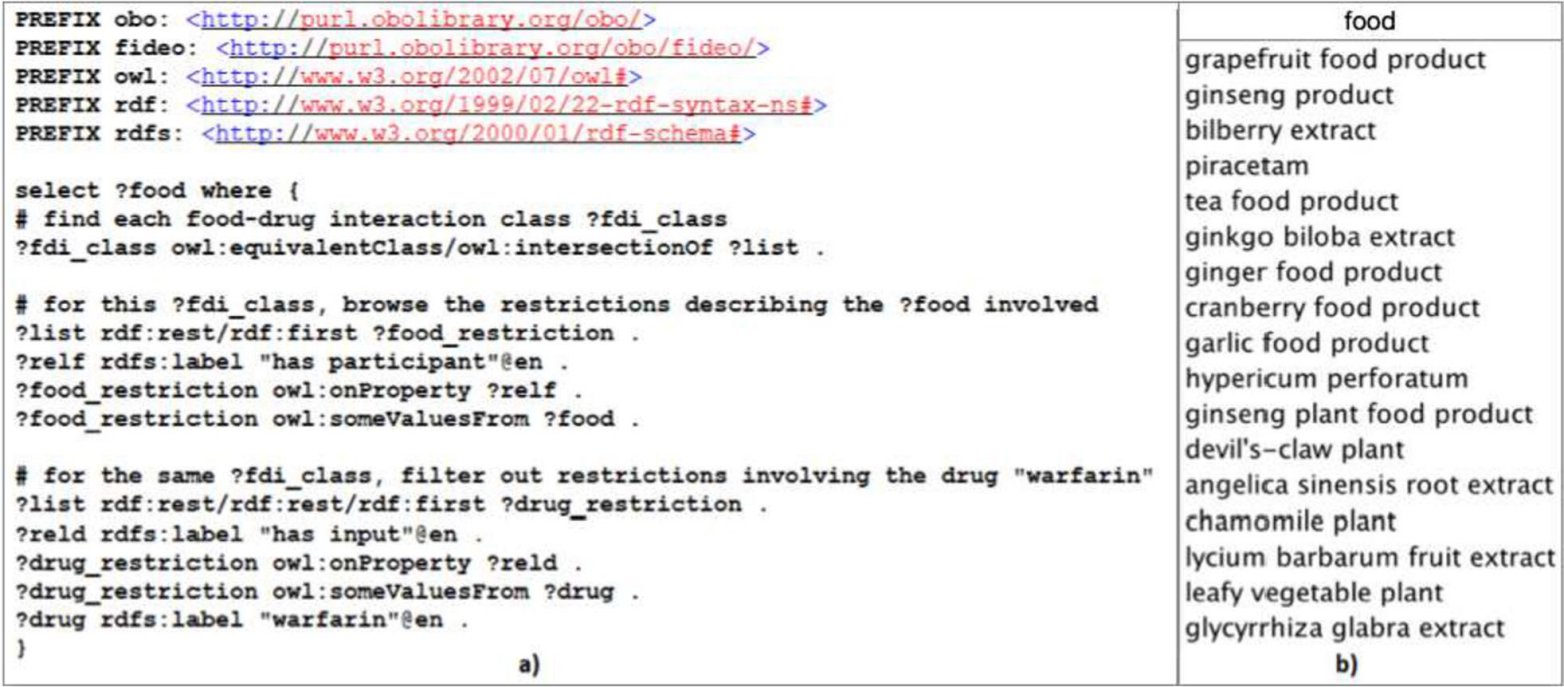
**a** SPARQL query over FIDEO to search for foods interacting with the drug “warfarin”, **b**) the 17 foods described as interacting with this drug in DrugBank and/or Hedrine

1. Panel a of Fig. 5 illustrates how it is possible to search for foods that interact with a given drug in FIDEO and panel b shows the 17 foods for which there is an interaction with the drug “warfarin”.
2. 46 drugs have been found as having an interaction with the “garlic food product”. The SPARQL query and the detailed list of drugs are available on GitHub [43].
3. Garlic interacts with 29 antithrombotic agents, including rivaroxaban and heparin, as can also be seen on GitHub. Of note, we performed an additional query showing that 31 foods are described as interacting with antithrombotic agents in FIDEO.
4. For the moment, we did not incorporate the type of interaction mechanism because this information is not always known and because of the complexities involved. It is noteworthy that when manually annotating foods in DrugBank sentences, we also evaluated whether each sentence mentioned an interaction mechanism or not (770 sentences out of the 3759 do). Examples of such sentences are: “*Alcohol increased peak serum concentrations of lithium*” for “lithium carbonate” and “*They* [Grapefruit products] *may interfere with warfarin metabolism and increase INR, increasing the risk of bleeding*” as shown for “warfarin” in Table 1.
5. Currently, we can partially answer this competency question because we have integrated in FIDEO the information regarding the knowledge source reporting the interaction (i.e. DrugBank or Hedrine) and the web page describing the food-drug interaction for DrugBank (this information not being available for Hedrine). Users can then search for articles mentioned in DrugBank and Hedrine that reported a given interaction. With CQ5, we can find that two Hedrine entries and a DrugBank Web page report an interaction between “garlic” and “warfarin”.
6. In the current version of FIDEO, it is not possible to provide the level of clinical importance of a food-drug interaction. However, since this information is available in Hedrine, users can find it indirectly. In addition, the fact that a given interaction is described in both DrugBank and Hedrine may also be an indicator of the confidence that can be placed in it.
7. Because we did not include food categories (for the sake of simplification), it is not yet possible to answer this competency question.
8. It is not possible to propose alternative drugs that can be taken to avoid the interactions because we have chosen to include in FIDEO only those drugs for which there is at least one interaction with food. However, this information can be retrieved by using the drug category to which the drug “warfarin” belongs (i.e. antithrombotic agents) and listing the drugs also belonging to this category in the ATC (note that it would be necessary to consider the finest ATC class to be sufficiently specific - “B01AA Vitamin K antagonists” in this case). However, it should be noted that such an alternative treatment recommendation must be validated by a healthcare professional.

## Discussion

### Findings

FIDEO currently represents 1702 food-drug interactions in a structured way. Of the eight competency questions originally identified in the creation of this ontology, three of them can now be fully addressed while one can be partially answered. In addition, the new version of FIDEO addresses the technical issues that were highlighted by the OBO dashboard [16] regarding the first version. The dashboard (and metrics) for each version of FIDEO is available on GitHub.

Regarding the methodology followed to enrich FIDEO, XOD has been very useful and will facilitate the management of the ontology evolution, especially if combined with ODK. Indeed, the use of ontology design patterns ensures a simple integration of new food-drug interactions. Moreover, the XOD methodology guarantees that FIDEO is aligned with reference ontologies to acquire knowledge that has already been described (and not reinvent the wheel), as recommended by the OBO Foundry [15].

Although not mentioned before in this article, FIDEO also respects the XOD 4 principle. It is indeed part of a global effort led by Damion Dooley to improve the interoperability of food-related ontologies, and in particular their articulation with FoodOn [21]. One of the authors (GB) actually participates in the Joint Food Ontology Workgroup [44], which meets monthly to address these issues.

### Limitations

When performing the manual annotation, we noticed a lack of homogenization in the textual descriptions of DrugBank food interactions. For example, with respect to tyramine-rich foods, DrugBank lists different sets of foods as follows:

- for “pargyline”: “*Foods that contain tyramine include yogurt, aged cheese, ripe bananas, wine, and sourdough bread*”,
- for “phenelzine”: “*Foods that contain tyramine include aged cheese, ripe bananas, red wine, some alcoholic beverages (beer), cured food, pickled food, and fava beans*”,
- for “linezolid”: “*Tyramine-containing foods include cheese, red wine, fava beans, pickled foods, cured foods, and alcoholic beverages*”.

An inherent limitation of this heterogeneity is that the interactions involving this category of foods have not been described systematically with the same foods. A possible solution to this problem is discussed in the next subsection.

Some of the modeling choices we have made can be discussed. Although pragmatic, the way food-drug interactions are described in FIDEO results in SPARQL queries that are not as straightforward as expected. This is partly due to the choice we made to represent these interactions as concepts in the ontology, rather than instances. However, we believe that this was a good way to proceed because the representation we have opted for allows us to make inferences about drug classes, in particular. On the other hand, there is one missing piece in our representation of food-drug interactions: the person or animal on which the interaction occurred. This information is not available in the resources we used to extract food-drug interactions, but it can probably be acquired from MeSH terms that index articles reporting such interactions.

In FIDEO, we have chosen to represent only proven food-drug interactions, but it might also have been useful to describe interactions for which it has been demonstrated that a given food has no impact on the effect of the associated drug. A simple solution for incorporating this type of knowledge would be to distinguish two different types of concepts: “non-harmful food-drug interaction” and “harmful food-drug interaction”. Finally, a given drug could have an impact on the nutritional component of foods (in this case, the *has_input* relationship should also have been used to describe the involvement of a food in the interaction), which we did not integrate in FIDEO because this information was not available in DrugBank and Hedrine.

### Further ontology enrichment

As highlighted in the findings regarding competency questions (Competency questions in FIDEO section), more work is needed. Future work we envisage to further enrich FIDEO includes incorporating knowledge about the different types of interaction mechanisms and integrating food categories, such as vitamin K-rich foods, antiplatelet/anticoagulant herbs or even herbal foods (which would bring together herbs currently classified as either food products or processed materials). Some of them can be found in FoodOn, such as fatty food (FoodOn:03305068) and plant food product (FoodOn:00001015), while others are not. These cases may involve more complex integration issues. We plan to report these missing food categories to the FoodOn curators so that they can enrich their ontology (if they consider that these notions are relevant to integrate), which would then facilitate the integration process in FIDEO.

To further enrich FIDEO, it would be interesting to use the DFI corpus that has been created recently [25]. This corpus may be broader than the one used when creating the first version of FIDEO since it also includes articles published in PubMed mentioning foods and drugs to which no MeSH term specifying a food-drug interaction is associated. From a broader, longer-term perspective, it could also be useful to integrate information on dietary supplements with known adverse effects and drug interactions, as described in particular in the iDISK knowledge base [45].

Finally, the link prediction theme is currently the subject of extensive research in the biomedical field, notably to predict food-drug interactions using deep learning [46] and graph mining approaches [47]. The objective of our work is different because we do not seek to predict previously unknown food-drug interactions but rather to represent them in a formal way so that users can automatically access structured information on interactions reported in the literature and online knowledge sources. However, predicted food-drug interactions could be incorporated in FIDEO as long as a confidence score is assigned to each food-drug interaction so that the interactions actually described in scientific articles can be distinguished from the predicted interactions.

## Data Availability

The datasets supporting the conclusions of this article are available at: https://gitub.u-bordeaux.fr/erias/fideo/.

## Abbreviations

ATC: Anatomical Therapeutic Chemical
BFO: Basic Formal Ontology
ChEBI: Chemical Entities of Biological Interest
CQ: Competency Question
DrOn: Drug Ontology
FAIR: Findable, Accessible, Interoperable, Reusable
FIDEO: Food Interactions with Drugs Evidence Ontology
IAO: Information Artifact Ontology
OBO: Open Biomedical and Biological Ontologies
OLS: Ontology Lookup Service
OWL: Web Ontology Language
ROBOT: ROBOT is an OBO Tool
XOD: eXtensible Ontology Development
